# Seasonal Genetic Drift of Human Influenza A Virus Quasispecies Revealed by Deep Sequencing

**DOI:** 10.3389/fmicb.2018.02596

**Published:** 2018-10-31

**Authors:** Cyril Barbezange, Louis Jones, Hervé Blanc, Ofer Isakov, Gershon Celniker, Vincent Enouf, Noam Shomron, Marco Vignuzzi, Sylvie van der Werf

**Affiliations:** ^1^Viral Populations and Pathogenesis, Department of Virology, Institut Pasteur, Paris, France; ^2^Molecular Genetics of RNA Viruses, Department of Virology, Institut Pasteur, Paris, France; ^3^UMR 3569, Centre National de la Recherche Scientifique, Paris, France; ^4^Cellule Pasteur, Université Paris Diderot–Université Sorbonne Paris Cité, Paris, France; ^5^Bioinformatics and Biostatistics HUB, The Center of Bioinformatics, Biostatistics and Integrative Biology, Institut Pasteur, Paris, France; ^6^Sackler Faculty of Medicine, Tel Aviv University, Tel Aviv, Israel

**Keywords:** influenza virus, quasispecies, NGS, genetic drift, influenza season

## Abstract

After a pandemic wave in 2009 following their introduction in the human population, the H1N1pdm09 viruses replaced the previously circulating, pre-pandemic H1N1 virus and, along with H3N2 viruses, are now responsible for the seasonal influenza type A epidemics. So far, the evolutionary potential of influenza viruses has been mainly documented by consensus sequencing data. However, like other RNA viruses, influenza A viruses exist as a population of diverse, albeit related, viruses, or quasispecies. Interest in this quasispecies nature has increased with the development of next generation sequencing (NGS) technologies that allow a more in-depth study of the genetic variability. NGS deep sequencing methodologies were applied to determine the whole genome genetic heterogeneity of the three categories of influenza A viruses that circulated in humans between 2007 and 2012 in France, directly from clinical respiratory specimens. Mutation frequencies and single nucleotide polymorphisms were used for comparisons to address the level of natural intrinsic heterogeneity of influenza A viruses. Clear differences in single nucleotide polymorphism profiles between seasons for a given subtype also revealed the constant genetic drift that human influenza A virus quasispecies undergo.

## Introduction

The development of deep sequencing technology, also known as next generation sequencing (NGS), offers a powerful tool to study the intrinsic heterogeneity of nucleic acids. For RNA viruses, it represents a major improvement in the study of their quasispecies nature over cloning and clone sequencing. The notion of quasispecies refers to the fact that RNA viruses exist as heterogeneous populations of closely related genetic variants (Domingo, 2006; Lauring and Andino, 2010), because their polymerase lacks fidelity and introduces point mutations during replication (Steinhauer et al., 1989a,b). Although the number of studies on RNA virus quasispecies evaluated by deep sequencing technology has dramatically increased in the past few years (Quinones-Mateu et al., 2014), there are still a limited number of such studies for influenza viruses.

Influenza A viruses belong to the *Orthomyxoviridae* viral family. One particularity of influenza A viruses is the segmented nature of their genome, which is composed of eight single-stranded RNA molecules of negative polarity. Each genomic segment encodes one major protein: the three polymerase sub-units PB2, PB1, and PA; the nucleoprotein (NP) associated with each of the RNA segments; the two envelope glycoproteins hemagglutinin (HA) and neuraminidase (NA); the matrix protein M1; and the multifunctional non-structural protein NS1. In addition, several segments encode one or more supplementary proteins through mechanisms of alternative splicing (PB2, MP/M, NS segments), alternative translation initiation (PB1, PA segments), or ribosomal frame-shift (PA segment), among which the ion channel protein M2 encoded by the M segment and the nuclear export protein NEP/NS2 encoded by the NS segment are best characterized (Wise et al., 2011; Jagger et al., 2012; Muramoto et al., 2013; Shaw and Palese, 2013; Yamayoshi et al., 2015). The two glycoproteins in the viral envelope, HA and NA, are used to define subtypes among influenza A viruses (Munster et al., 2007; Tong et al., 2013). Continuous genetic variation of influenza viruses due to the low fidelity of the viral polymerase may translate into antigenic variations through mutations in the HA and/or NA, a phenomenon also known as antigenic drift. In addition, introductions into the human population of new influenza A viruses, or antigenic shift, can occur through cross-species transmission from the animal reservoir (mainly poultry and pigs), and occasionally lead to devastating pandemics. After a pandemic wave, the newly introduced viruses gradually become seasonal (Tscherne and Garcia-Sastre, 2011). They are then responsible for yearly epidemics, mostly causing a relatively mild disease of the upper respiratory tract, that nonetheless represent a major burden for public health worldwide, with significant morbidity and mortality (Molinari et al., 2007; Ortega-Sanchez et al., 2012). Since 1977, two influenza A virus subtypes, H1N1 and H3N2, co-circulate in the human population. While undergoing genetic variation, H1N1 viruses with the H275Y mutation in NA that confers resistance to antiviral oseltamivir treatment emerged in late 2007 in the human population and circulated independently of the use of antiviral treatment to eventually become dominant over the sensitive H1N1 viruses by late 2008 (Meijer et al., 2009; Yang et al., 2011). These H1N1 viruses were fully replaced by the H1N1pdm09 viruses that originated from pigs and resulted in the 2009 pandemics (Neumann et al., 2009). The H1N1pdm09 viruses became established in the human population and currently co-circulate with H3N2 viruses.

Deep sequencing technology for influenza virus has mainly been used to determine the consensus sequence more rapidly than conventional methods and for a large number of samples simultaneously (Hoper et al., 2011; Ren et al., 2013; Rutvisuttinunt et al., 2013; Lin et al., 2014; Monne et al., 2014; Lee et al., 2016; Zhao et al., 2016), and to study the intrinsic heterogeneity of the virus genome on cell-adapted viruses (Watson et al., 2013; Thyagarajan and Bloom, 2014) or in clinical samples but focusing primarily on the HA and NA genes (Chen et al., 2010; Ghedin et al., 2011; Fordyce et al., 2013; Téllez-Sosa et al., 2013; Pizzorno et al., 2014; Dinis et al., 2016; Poon et al., 2016; Pichon et al., 2017). Deep sequencing was also applied to monitor the emergence of antiviral resistance in patients treated with oseltamivir (Ghedin et al., 2012; Rogers et al., 2015) or to understand transmission pathways and investigate local outbreaks (Ghedin et al., 2012; Poon et al., 2016; Meinel et al., 2018). Here, by evaluating the virus quasispecies diversity directly in infected human respiratory specimens, we demonstrated differences in the intrinsic genetic diversity between subtypes and showed that the composition of the quasispecies evolves season after season.

## Results

### Sample Characteristics

The samples (Supplementary Table 1) used in this study were collected during five consecutive seasons of influenza surveillance by the Northern France National Influenza Center (from 2007–2008 to 2011–2012). Respiratory specimens were selected from the collection of samples that tested positive for influenza A virus and for which the virus subtype was determined. For each season, more samples were selected from the dominant subtype that was circulating: pre-pandemic H1N1 (sH1N1) in 2007–2008, H3N2 (sH3N2) in 2008–2009 and 2011–2012, and pandemic H1N1pdm09 (pH1N1) in 2009–2010 and 2010–2011. For sH1N1 positive samples, selection was also based on the presence or absence of the H275Y mutation in NA, which confers resistance to oseltamivir. In addition, the selection included samples from mild cases collected through the GROG community surveillance network, and from severe cases defined as requiring external respiratory assistance and collected through the RENAL network of hospital laboratories. The median patient age was 22, 41, and 29 years old for sH1N1, H3N2 and pH1N1, respectively (95% CI of median: 5–38, 30–50, and 16–36 years old, respectively), with the patient age ranging from a minimum of 2, 0.8, and 3 years-old to a maximum of 81, 91, and 83 years old, respectively. The interval from onset of symptoms to sample collection was similar for the three subtypes, with a median interval of 1, 1.5, and 1 day for sH1N1, H3N2, and pH1N1, respectively (95% CI of median: 1–2 days for the three subtypes). The maximum sampling time from symptoms onset was 9, 5, and 6 days for sH1N1, H3N2, and pH1N1, respectively. PCRs were performed on cDNA synthesized from RNA directly extracted from the clinical specimen in order to avoid artifacts due to virus amplification in cell culture or in eggs. The strategy implemented to cover as much as possible of the coding regions was based on 12 specific PCRs, the four larger segments being amplified by two overlapping PCRs. Moreover, high-fidelity enzymes for both cDNA synthesis and PCR were used to reduce the incorporation of errors during viral genome amplification.

### Read Quality Control and Read Cleaning

For each sample, reads obtained by Illumina sequencing were checked for quality (Supplementary Table 1) and cleaned by three successive steps using FastQC and fqcleaner Galaxy tools (Mareuil et al., 2017): first, reads were cleaned of Illumina adaptors and based on Phred scores (retained bases had a score above 30); then reads were cleaned of PCR primer sequences and non-PCR target contaminants; finally the remaining reads were cleaned of non-influenza virus contaminants, using the sequence of other viruses manipulated in the laboratory. For all samples, this last cleaning showed that very few contaminations occurred during sample preparation. For good runs, between 60 and 70% of the reads were retained after the three steps of cleaning. One run for pH1N1 was characterized by an excess of Illumina adaptor contaminants, but the remaining cleaning steps led to proportions of retained reads similar to what was observed for good runs, even if the overall retain proportion was of course lower. The reads obtained after the three steps of cleaning were deposited in the European Nucleotide Archive database for NGS sequences^1^ (Accession No. ERP012790). The consensus sequence was extracted for each segment of each sample and was deposited in the Global Initiative on Sharing All Influenza Data (GISAID) database (accession numbers in Supplementary Table 1).

### Comparison of ViVAN and LoFreq Pipelines to Analyze Virus NGS Data

To analyze the intrinsic genetic diversity, two pipelines were used to process the cleaned reads and identify positions with significant variants, using each sample’s own consensus sequence as reference. Both pipelines were developed by independent groups and use different algorithms to identify significant variants. The ViVAN pipeline is an all-inclusive pipeline (its own mapper is included) and was recently developed specifically for virus NGS data (Isakov et al., 2015). LoFreq was also specifically designed for virus NGS data and is already recognized within the scientific community (Wilm et al., 2012); it was used in conjunction with Bowtie mapper but we will refer to “LoFreq pipeline” for the whole process of mapping and extracting variant positions.

**FIGURE 1:**
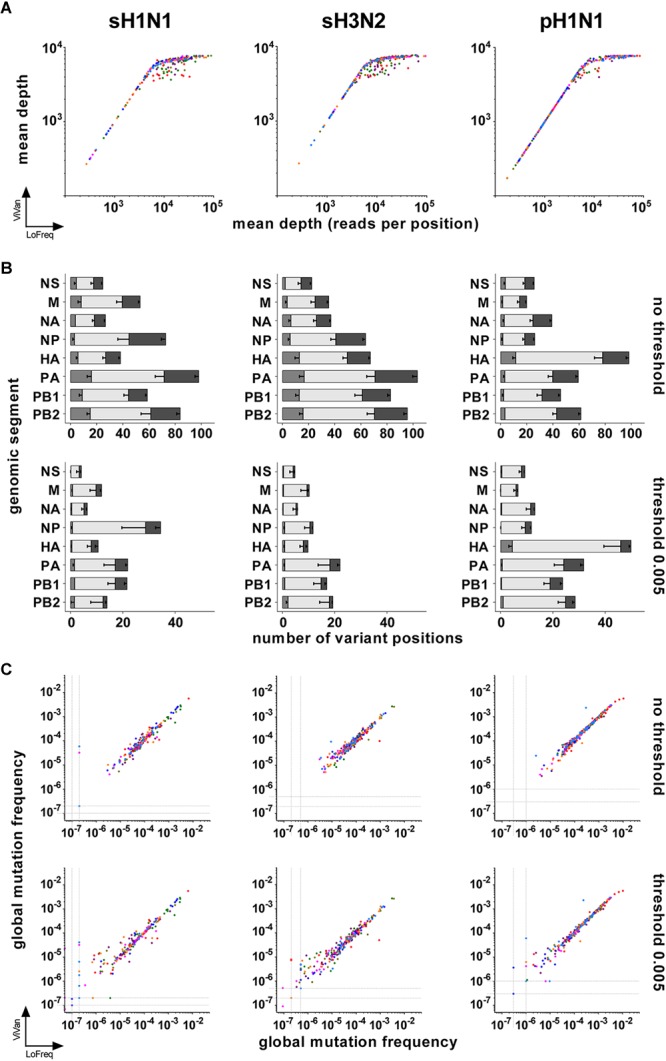
Comparison of LoFreq and ViVAN. **(A)** Correlation of the mean depths of sequencing (in number of reads per position) obtained by both pipelines. *X*-axis: LoFreq; *Y*-axis: ViVAN. Each dot represents one segment of one sample: polymerase sub-unit PB2: olive; polymerase sub-unit PB1: green; polymerase sub-unit PA: purple; hemagglutinin HA: red; nucleoprotein NP: dark blue; neuraminidase NA: pink; matrix and ion channel proteins M: orange; non-structural protein and nuclear export protein NS: light blue. **(B)** Mean number of variant positions identified by the pipelines, without or with setting a mutation frequency threshold. For each bar, from left to right: gray, specific to LoFreq; light gray, common to LoFreq and ViVAN; dark gray, specific to ViVAN. Error bars represent the Standard Error to the Mean. **(C)** Correlation of the global mutation frequencies obtained with both pipelines with or without setting a mutation frequency threshold. *X*-axis: LoFreq; *Y*-axis: ViVAN. Each dot represents one segment of one sample: PB2: olive; PB1: green; PA: purple; HA: red; NP: dark blue; NA: pink; M: orange; NS: light blue.

We first compared the “depth of sequencing” obtained by both pipelines. Mapping of the cleaned reads on the reference sequence allowed to extract the number of times each position was covered, i.e., the depth of sequencing. For both pipelines, it was found to be homogeneous along each PCR product. The mean depth of sequencing was calculated for each viral genomic segment sequence of each sample and ranged from around 200 to around 10^5^ reads per position, with the majority of the mean depth values being above 10^3^ reads per position. Both pipelines gave similar results for a given sample, except above 10^4^ reads per position where the LoFreq pipeline was more powerful to map cleaned reads (Figure 1A and Supplementary Table 1). Consequently, the median of the mean depth values was higher with LoFreq (in reads per position: for sH1N1, median 11989, 95% CI of median 9894–14065, min 269, max 89267; for sH3N2, median 11567, 95% CI of median 10237–12293, min 273, max 94094; for pH1N1, median 3790, 95% CI of median 2816–4781, min 175, max 110570) than with ViVAN (in reads per position: for sH1N1, median 6228, 95% CI of median 5796–6471, min 268, max 7747; for sH3N2, median 6398, 95% CI of median 6034–6703, min 271, max 7633; for pH1N1, median 3698, 95% CI of median 2785–4343, min 173, max 7751).

Both pipelines include tests that take into account the depth of sequencing, the complementarity of read orientation, the error rate of the Illumina method and the nature of the nucleotides in the sequence surrounding the position of interest, in order to identify positions with significant variants, even if those are present at a very low frequency (for a given position, number of times a base is different from the reference sequence – here the consensus sequence of the given sample – divided by depth of sequencing). It is commonly accepted that very low frequency variants might be unreliable. We compared the numbers of significant variants obtained by both pipelines without a threshold or after setting a mutation frequency threshold at 0.005 for retaining significant variants (meaning that only positions with a significant variant representing more than 0.5% of the reads would be counted). This threshold represents five times the error rate recognized for the Illumina method using standard, non-high-fidelity enzymes to prepare PCR products from RNA (Nakamura et al., 2011). While an important number of variant positions were specific of each pipeline when no threshold was set, Figure 1B shows that a 0.005 frequency threshold reduced the number of pipeline-specific variant positions (Supplementary Tables 1, 2). This was particularly true for the LoFreq pipeline, for which only a few pipeline-specific variants remained for the three subtypes.

We further estimated the viral intrinsic genetic diversity by calculating, for each variant caller pipeline, the “global mutation frequency” for each segment of each sample, using the data generated by the ViVAN and LoFreq pipelines when no threshold or a 0.005 threshold was used (Supplementary Table 1). Basically, the number of variant bases corresponding to all identified or retained variant positions was divided by the total number of sequenced bases (i.e., the sum of the depth of sequencing) covering a given item (segment-sample). When there was no variant identified in a segment (especially when the 0.005 threshold was applied), we allocated an arbitrary value for global mutation frequency slightly below the lowest calculated value among the samples of the same group (one genomic segment of one subtype), in order to avoid null global mutation frequencies. A good correlation between the two pipelines was found when the global mutation frequencies were compared (Figure 1C and Table 1). No correlation was found between possible biases (mean depth, age of patient, virus load) and the global mutation frequency (Supplementary Figure 1 and Supplementary Table 3).

**Table 1:** Correlation between global mutation frequencies obtained with LoFreq and ViVAN, evaluated by Spearman non-parametric test.

Virus	Threshold	Genomic segment	Pairs	Spearman correlation test	
				rs	95% CI
sH1N1	0.0	PB2	27	0.9695	0.9318–0.9865
		PB1	27	0.9811	0.9574–0.9917
		PA	27	0.9536	0.8973–0.9794
		HA	27	0.9470	0.8832–0.9764
		NP	27	0.9707	0.9344–0.9870
		NA	27	0.9690	0.9307–0.9863
		M	26	0.9096	0.8020–0.9600
		NS	22	0.9367	0.8476–0.9744
	0.5	PB2	27	0.9858	0.9679–0.9937
		PB1	27	0.9750	0.9439–0.9889
		PA	27	0.8630	0.7129–0.9375
		HA	27	0.9321	0.8516–0.9696
		NP	27	0.9807	0.9565–0.9915
		NA	27	0.9670	0.9263–0.9854
		M	26	0.9304	0.8456–0.9694
		NS	22	0.9133	0.7948–0.9647
pH1N1	0.0	PB2	38	0.9941	0.9883–0.9970
		PB1	35	0.9950	0.9897–0.9975
		PA	39	0.9921	0.9845–0.9959
		HA	39	0.9883	0.9771–0.9940
		NP	38	0.9932	0.9866–0.9966
		NA	39	0.9954	0.9910–0.9976
		M	39	0.9860	0.9728–0.9928
		NS	39	0.9775	0.9564–0.9885
	0.5	PB2	38	0.9965	0.9931–0.9982
		PB1	35	0.9988	0.9976–0.9994
		PA	39	0.9860	0.9727–0.9928
		HA	39	0.9931	0.9866–0.9965
		NP	38	0.9928	0.9859–0.9964
		NA	39	0.9945	0.9893–0.9972
		M	39	0.9880	0.9767–0.9939
		NS	39	0.9642	0.9310–0.9815
sH3N2	0.0	PB2	35	0.9549	0.9101–0.9776
		PB1	35	0.9826	0.9649–0.9915
		PA	35	0.9504	0.9014–0.9754
		HA	35	0.9437	0.8884–0.9720
		NP	35	0.9894	0.9784–0.9948
		NA	35	0.9731	0.9459–0.9867
		M	35	0.9717	0.9431–0.9860
		NS	32	0.9065	0.8120–0.9547
	0.5	PB2	35	0.9661	0.9320–0.9832
		PB1	35	0.9694	0.9386–0.9849
		PA	35	0.9569	0.9139–0.9786
		HA	35	0.9383	0.8779–0.9693
		NP	35	0.9874	0.9744–0.9938
		NA	35	0.9837	0.9671–0.9920
		M	35	0.9935	0.9868–0.9968
		NS	32	0.9781	0.9542–0.9896

### Group Comparisons: Severity Status Within Subtype; Seasons Within Subtype; Between Subtypes

**FIGURE 2:**
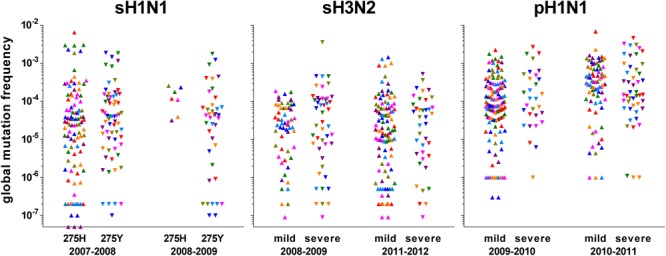
Representation of the global mutation frequencies according to season and group of interest. Values were calculated from the data obtained with LoFreq at a 0.005 threshold. Each symbol represents one segment of one sample: polymerase sub-unit PB2: olive; polymerase sub-unit PB1: green; polymerase sub-unit PA: purple; hemagglutinin HA: red; nucleoprotein NP: dark blue; neuraminidase NA: pink; matrix and ion channel proteins M: orange; non-structural protein and nuclear export protein NS: light blue. For pre-pandemic sH1N1 viruses, groups were based on the oseltamivir-resistance mutation H275Y (275H: sensitive; 275Y: resistant) in the neuraminidase; for both H3N2 and pandemic pH1N1 viruses, groups were based on the severity according to the network of sampling (mild through community physicians, severe through hospitals).

Global mutation frequency data generated with the LoFreq pipeline at a 0.005 threshold were used to analyze the differences between different groups. Mann–Whitney–Wilcoxon non-parametric test was used for all pairwise comparisons. Three subtypes were considered: sH1N1, sH3N2, and pH1N1. Within sH1N1, subgroups were defined, based on the mutation conferring resistance to oseltamivir (H275Y in the neuraminidase) and on the season. Within sH3N2 and pH1N1, subgroups were defined, based on the severity status (mild and severe cases) and on the season. The few samples of the non-dominant subtype during a season were not included and only the samples belonging to the specified groups within a season were used for comparison. The global mutation frequencies calculated for each segment of each sample of each group are shown in Figure 2.

#### Comparisons of Global Mutation Frequency Distributions Within Each Subtype

Whatever the genomic segment, no significant difference in the global mutation frequency distribution between any subgroups was observed for sH1N1 samples; they were pooled to form a unique group “sH1N1.” The median global mutation frequency for sH1N1 ranged from 6.8 mutations per 10^6^ bases for the NS segment to 1.4 mutations per 10^4^ bases for the PB1 segment (Table 2). Similarly for sH3N2, whatever the genomic segment, no significant difference was observed in the global mutation frequency distribution between the mild and severe cases or between the two studied seasons; all samples were pooled to form the “sH3N2” group. The median global mutation frequency for sH3N2 ranged from one mutation per 10^5^ bases for the NA segment to 3.2 mutations per 10^5^ bases for the NP segment (Table 2). For pH1N1, whatever the genomic segment, no significant difference was observed between mild and severe cases in each studied season. When comparing seasons, a significant difference was observed in the global mutation frequency distribution for genomic segment PA encoding the PA polymerase sub-unit (Mann–Whitney–Wilcoxon *U* = 61; N_2009-2010_ = 20; N_2010-2011_ = 16; actual difference = 2.6 × 10^-4^; 95% CI of difference = 6.4 × 10^-5^ to 8.9 × 10^-4^) and for segment MP encoding the M1 matrix and M2 ion channel proteins (Mann–Whitney–Wilcoxon *U* = 95; N_2009-2010_ = 20; N_2010-2011_ = 16; actual difference = 1.9 × 10^-4^; 95% CI of difference = 0 to 3.9 × 10^-4^). The samples were pooled to form the “pH1N1” group, except for PA and MP, for which each season was kept separated for the subtype comparison. The pH1N1 median global mutation frequency ranged from 6.1 mutations per 10^5^ bases for the MP segment of season 2009–2010 to 4.2 mutations per 10^4^ bases for the PB2 segment encoding the PB2 polymerase sub-unit (Table 2).

**Table 2:** Median global mutation frequency per segment for each virus.

Virus	Genomic segment	Specific season	Sample number	Median	95% CI of median	
					Actual %	Lower to upper
sH1N1	PB2		27	2.5 × 10^-5^	98.08	6.4 × 10^-6^ to 5.0 × 10^-5^
	PB1		27	1.4 × 10^-4^	98.08	3.5 × 10^-5^ to 8.3 × 10^-4^
	PA		27	2.9 × 10^-5^	98.08	1.1 × 10^-5^ to 4.0 × 10^-5^
	HA		27	5.1 × 10^-5^	98.08	9.4 × 10^-6^ to 1.2 × 10^-4^
	NP		27	4.0 × 10^-5^	98.08	9.9 × 10^-6^ to 2.1 × 10^-4^
	NA		27	4.3 × 10^-5^	98.08	1.6 × 10^-5^ to 1.1 × 10^-4^
	M		26	2.3 × 10^-5^	97.10	7.0 × 10^-6^ to 8.2 × 10^-5^
	NS		22	6.7 × 10^-6^	98.31	2.0 × 10^-7^ to 6.3 × 10^-5^
sH3N2	PB2		31	2.3 × 10^-5^	97.06	7.0 × 10^-6^ to 7.5 × 10^-5^
	PB1		31	2.9 × 10^-5^	97.06	9.4 × 10^-6^ to 8.3 × 10^-5^
	PA		31	2.0 × 10^-5^	97.06	7.8 × 10^-6^ to 1.1 × 10^-4^
	HA		31	2.5 × 10^-5^	97.06	8.0 × 10^-6^ to 6.2 × 10^-5^
	NP		31	3.2 × 10^-5^	97.06	1.8 × 10^-5^ to 6.7 × 10^-5^
	NA		31	1.0 × 10^-5^	97.06	3.0 × 10^-6^ to 5.3 × 10^-5^
	M		31	1.9 × 10^-5^	97.06	3.8 × 10^-6^ to 6.7 × 10^-5^
	NS		28	2.3 × 10^-5^	96.43	9.0 × 10^-6^ to 4.5 × 10^-5^
pH1N1	PB2		35	4.2 × 10^-4^	95.90	1.0 × 10^-4^ to 5.4 × 10^-4^
	PB1		32	1.7 × 10^-4^	97.99	6.1 × 10^-5^ to 7.0 × 10^-4^
	PA	2009–2010	20	6.9 × 10^-5^	95.86	3.9 × 10^-5^ to 2.3 × 10^-4^
		2010–2011	16	3.3 × 10^-4^	97.87	1.0 × 10^-4^ to 1.2 × 10^-3^
	HA		36	1.7 × 10^-4^	97.12	6.4 × 10^-5^ to 5.4 × 10^-4^
	NP		35	1.3 × 10^-4^	95.90	5.9 × 10^-5^ to 2.6 × 10^-4^
	NA		36	1.1 × 10^-4^	97.12	5.6 × 10^-5^ to 2.8 × 10^-4^
	M	2009–2010	20	6.1 × 10^-5^	95.86	1.0 × 10^-6^ to 1.6 × 10^-4^
		2010–2011	16	2.5 × 10^-4^	97.87	2.2 × 10^-5^ to 7.6 × 10^-4^
	NS		36	1.3 × 10^-4^	97.12	6.6 × 10^-5^ to 2.9 × 10^-4^

#### Comparisons of Global Mutation Frequency Distributions Between Subtypes

**FIGURE 3:**
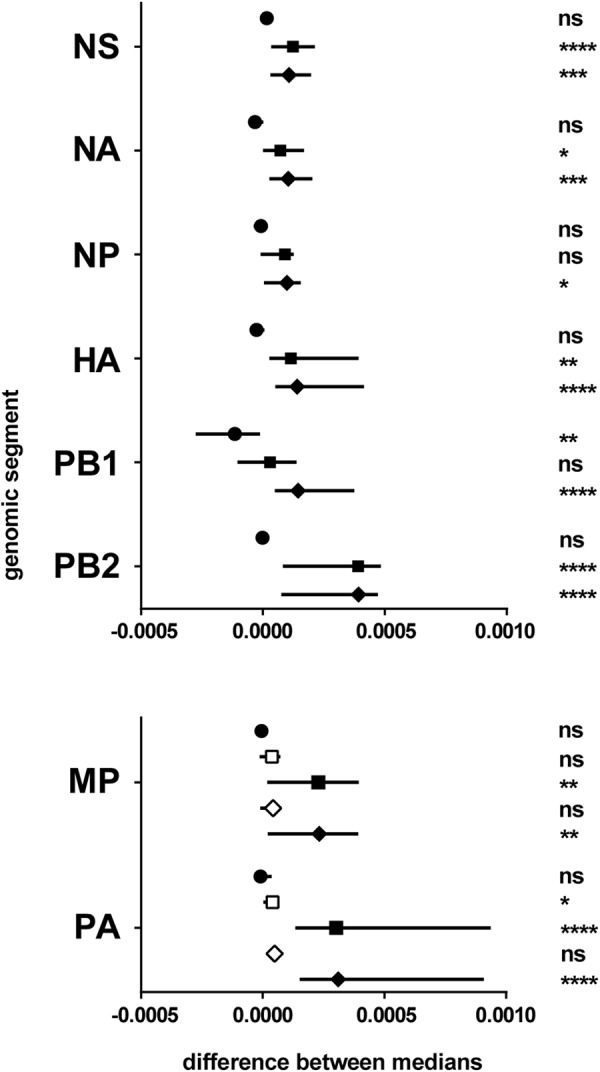
Differences between subtypes. Results of the pairwise comparisons by Mann–Whitney–Wilcoxon test of the global mutation frequency distributions (Supplementary Table 4). The genomic segments considered are mentioned on the left. *X*-axis: difference between the medians (symbol) and 95% CI of the difference (line). Symbols: circle, sH1N1 vs. sH3N2; square, sH1N1 vs. pH1N1 (for MP and PA, open square for pH1N1 season 2009–2010, plain square for pH1N1 season 2010–2011); diamond, sH3N2 vs. pH1N1 (for MP and PA, open square for pH1N1 season 2009–2010, plain square for pH1N1 season 2010–2011). ns, non-significant; ^∗^0.05 ≥*p*-value > 0.01; ^∗∗^0.01 ≥*p*-value > 0.001; ^∗∗∗^0.001 ≥*p*-value > 0.0001; ^∗∗∗∗^*p*-value ≤ 0.0001.

No significant difference in the global mutation frequency distribution was observed between sH1N1 and sH3N2, except for the PB1 polymerase sub-unit genomic segment. On the contrary, pH1N1 was found significantly different from both sH1N1 and sH3N2 for most genomic segments (Figure 3 and Supplementary Table 4). The pH1N1 subtype clearly differed from both sH1N1 and sH3N2 subtypes, and was found to be more heterogeneous with higher global mutation frequencies in most genomic segments.

#### Comparisons of vSNP Pattern Between Seasons

**FIGURE 4:**
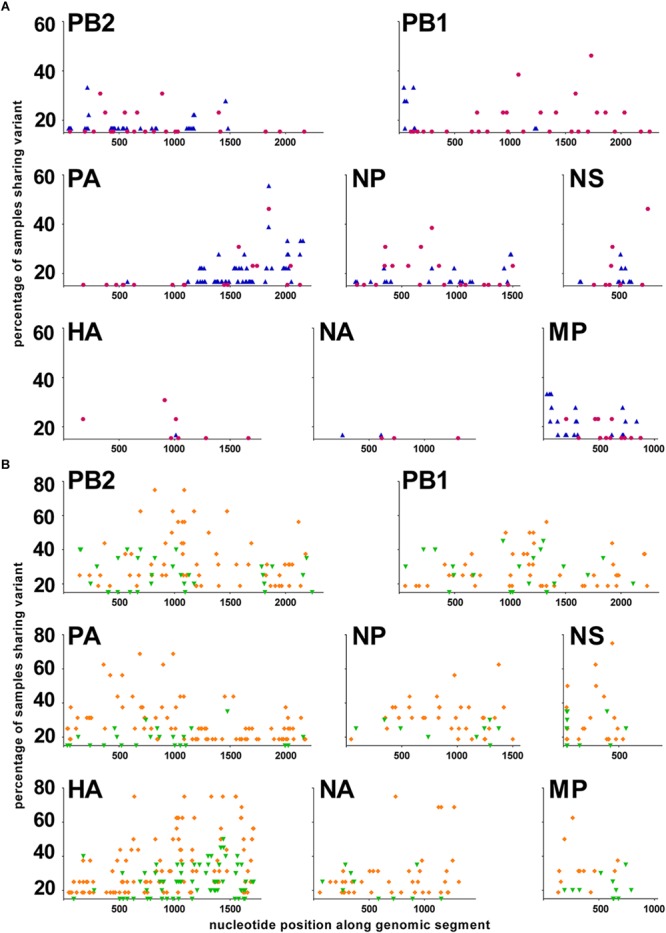
Distribution of vSNPs along the genome. A viral Single Nucleotide Polymorphism (vSNP) is a position with a significant variant that has been identified in at least 15% of the samples in a group. *X*-axis: nucleotide position along each genomic segment; *Y*-axis: percentage of samples sharing a significant variant. **(A)** sH3N2 samples. In purple: season 2008–2009; in blue: season 2011–2012. **(B)** pH1N1 samples. In green: season 2009–2010; in orange: season 2010–2011.

To further analyze the quasispecies structure for the different viruses, we examined the distribution of the positions with a significant variant along each of the genomic segments. A viral single nucleotide polymorphism (vSNP) was defined as a significant variant position that was shared by more than 15% of the samples within a subtype, within a given season. Differences in the vSNP patterns were observed between the two studied seasons for both sH3N2 and pH1N1 subtypes (Figure 4). Subtype sH3N2 was characterized by very few vSNPs in the HA and NA segments, and vSNPs in the other segments that corresponded to variants that were mainly shared by less than 40% of the samples (with the exceptions of position 1731 in the PB1, 1849 in the PA, and 761 in the NS segments). Subtype pH1N1 was characterized by many vSNPs in the HA and NA segments. During season 2009–2010, most vSNPs were variants that were shared by less than 50% of the samples (Figure 4B, green). During season 2010–2011, several vSNPs in PB2, PA, HA, NP, NA, M, and NS segments were variants shared by 50–80% of the samples, whereas most vSNPs in the PB1 segment were shared by less than 50% of the samples (Figure 4B, orange).

## Discussion

The intrinsic heterogeneity of nearly the entire viral genome of influenza A viruses was evaluated directly in respiratory specimens collected from a large number of infected patients over five consecutive seasons in a defined geographic area (Northern half of France). Depth of sequencing was not only high, with a coverage largely above 1000 for most PCR products, but it was also found to be extremely homogeneous along the PCR products, which represented an improvement compared to some published data (Kuroda et al., 2010; Kampmann et al., 2011; Lin et al., 2014; Welkers et al., 2015). Differences in the library generation protocols probably explain those results and highlight the fact that many parameters are still not totally controlled when deep sequencing technology is applied (Beerenwinkel et al., 2012). The use of high fidelity enzymes for both the reverse transcription and the PCR steps allowed to limit the introduction of errors during the viral genome amplification, with an estimated overall error reduction by 150 times compared to classical enzymes according to the manufacturers’ data. The introduction of errors during the preparation of libraries for deep sequencing runs is a well-known limitation of NGS that needs to be taken into account (Willerth et al., 2010; Acevedo and Andino, 2014). An 0.001 mutation frequency has been used by others as a threshold to define viral subpopulations based on Illumina technology results (Nakamura et al., 2011). We decided to use, for specific comparisons, only positions with a mutation frequency above 0.005 and that have been identified by two independent pipelines that were specifically designed for the identification of virus quasispecies, making our results extremely reliable and comparable to previous studies (Welkers et al., 2015). However, the physiological relevance of low mutation frequencies is still unknown and it is thus difficult to determine the impact of any subpopulations described by deep sequencing.

The global mutation frequency that we calculated for the influenza virus samples appeared slightly lower than the published data on RNA virus polymerase error-rates (Parvin et al., 1986; Drake and Holland, 1999; Sanjuan et al., 2010), suggesting that some bottleneck events might occur between replication of the genome in the cells of the respiratory tract and excretion of the virus as collected in respiratory specimens (Xue et al., 2018). The fact that no bias was observed demonstrated that the level of heterogeneity detected by deep sequencing was not correlated to the virus load in the sample or to the age of the patient. This is an important point, as one could have hypothesized that the virus variability could be higher in infected children or that a higher mutation frequency could artificially be the consequence of more virus genomes being present in the sample. Deep sequencing approaches based on clinical samples instead of cell culture-amplified virus to evaluate the viral quasispecies have long focused on HIV and hepatitis viruses (Quinones-Mateu et al., 2014). Most studies concerned the appearance of resistance and the evolution of specific related mutations under antiviral treatments (Simen et al., 2009; Nasu et al., 2011; Fisher et al., 2012; Nishijima et al., 2012; Svarovskaia et al., 2012; Li et al., 2013; Rodriguez et al., 2013). A few studies were in experimental *in vivo* conditions, using animal models (Moncla et al., 2013; Sutton et al., 2014), and some focused on the intrinsic heterogeneity of the virus as it is excreted, following, for example, HIV1/HCV quasispecies in the semen of naturally (co-)infected males (Paranjpe et al., 2002; Briat et al., 2005). The importance of studying the quasispecies composition of RNA viruses was recently highlighted when deep sequencing analysis allowed to follow the virus evolutionary trajectories upon the emergence of a Chikungunya virus variant responsible for an epidemic in the Indian Ocean in 2006 (Stapleford et al., 2014).

The description of vSNPs common to several samples within a subtype has been previously reported for influenza viruses mainly at positions involved in resistance to antivirals (Téllez-Sosa et al., 2013; Pizzorno et al., 2014). In the present study, vSNPs in the whole genome could be identified because a relatively large number of samples were analyzed for each subtype and for two seasons for sH3N2 and pH1N1. The physiological importance of the vSNPs we found in the different genomic segments is totally unknown and will require further investigation with the help of reverse genetics systems. Most of the positions with vSNPs concerned the third nucleotide of codons, and the variant mutations were consequently mainly synonymous (Supplementary Table 5), meaning that the phenotype of the variant subpopulations was probably not different from that of the main subpopulation. If intrinsic genetic variability does not confer variability in phenotypes, i.e., potentially immediate fitness advantages (Debbink et al., 2017; McCrone et al., 2017), it must have an importance in terms of evolution and adaptability. A heterogeneous population, even with variants at very low frequency, could facilitate or speed up evolution. Comparison of two seasons for a given subtype strikingly highlighted the evolution dynamics and quasispecies plasticity of influenza A viruses. Whereas no difference was observed in the global mutation frequency distribution between the two seasons for both sH3N2 and pH1N1, the vSNPs dispersal along the genome clearly showed differences between the two seasons.

Interestingly, contrary to sH3N2 for which only a few positions were vSNPs in the HA and NA gene sequences, pH1N1 was characterized by the presence of many vSNPs in the glycoprotein gene sequences. Together with the higher level of global heterogeneity compared to sH1N1 and sH3N2, and despite the relative stability at the consensus sequence level since its introduction in human in 2009 (Klein et al., 2014), it demonstrated that pH1N1, a virus recently introduced in the human population, is still adapting to the human host, exploring different areas of the sequence space. This plasticity of the quasispecies was nonetheless also observed for sH3N2 viruses, which have been circulating in the human population since the late 1960s, since vSNP differences were identified between seasons. This phenomenon was nonetheless less pronounced than for pH1N1, but it illustrated the permanent, underlying genetic drift occurring in human influenza viruses.

Two intriguing observations will require further investigations. First, for both pH1N1 and sH3N2, non-synonymous vSNPs were identified in viral components involved in intracellular steps of the virus replication, such as the polymerase sub-units, the nucleoprotein, the matrix M1 protein, and the non-structural NS1 protein. We hypothesize that these vSNPs might provide some flexibility in different processes, either directly by modulating the activity of a given protein, or indirectly by affecting the interaction with cellular factors. The second point concerns the main determinants of influenza virus antigenic evolution: the envelope glycoproteins HA and NA. It is unclear why so few vSNPs were identified in the HA and NA for sH3N2. We wonder whether a similar situation would be observed for sH3N2 samples collected during a season when sH3N2 does not dominate and whether there could be a relationship between the level of intrinsic heterogeneity and the alternate dominance observed between subtypes. We also noticed that several variants that were identified as minority mutations in the samples used in this study were later detected as dominant in other parts of the world and used to define some genetic clades/groups. Thus, for pH1N1, mutations A134T/S183P in the HA and Q313R/V394I in the NA, that we identified as minority mutations during the 2010–2011 season, were used the following season by WHO Collaborating Centers (Barr et al., 2014) to define group 3 sequences (represented by A/Hong-Kong/3934/2011). Similarly, mutations S143G/A197T in the HA and N44S in the NA were used to define group 7 sequences (represented by A/St-Petersburg/100/2011).

Studying the intrinsic heterogeneity of influenza A viruses by deep sequencing directly from respiratory specimens clearly gave interesting insights into the virus evolution dynamics by adding a new dimension to analyses performed on consensus sequences. It would now be interesting to process samples from more epidemic seasons to better describe the phenomenon of evolutionary plasticity. Studies by other groups have not been able to address this question due to the limited number of seasons (Dinis et al., 2016; Poon et al., 2016) or samples (McCrone et al., 2017) that were under consideration or available. Thus, analyzing future seasons of pH1N1 dominance would show if the virus would maintain its high level of heterogeneity and continue to explore the sequence space, or if some equilibrium might soon be reached with a level of heterogeneity similar to that observed for sH3N2. Given the large dispersion observed in the global mutation frequency distribution within a group, it nonetheless seems necessary to process a large set of samples in order to identify statistically significant differences; this will be more achievable as the NGS technologies evolve and the cost decreases.

## Materials and Methods

### Clinical Samples

Samples were selected among influenza A virus-positive respiratory specimens available in the collection of the National Influenza Centre (NIC) for Northern France. For yearly epidemiological surveillance of influenza viruses circulating during seasonal epidemics, nasopharyngeal swabs were regularly collected by physicians of the GROG network from patients with ARI (acute respiratory illness) and sent to the NIC for virus detection and characterization. As part of its routine surveillance activities, the NIC also received respiratory specimens (nasopharyngeal swabs or nasopharyngeal aspirates) from the hospital laboratories belonging to the RENAL network, which mainly sent samples from hospitalized patients. In this study, for H3N2 (sH3N2) and pandemic 2009 H1N1 (pH1N1), mild cases were defined as patients from the community sampled by the GROG network, while severe cases were patients with severe acute respiratory distress requiring external respiratory assistance and were sampled by the RENAL network. For pre-pandemic H1N1 (sH1N1), sensitivity and resistance to Oseltamivir were based on the amino acid nature at position 275 in the neuraminidase NA protein (H and Y, respectively). All specimens were declared to the Ministère de l’Enseignement Supérieur et de la Recherche (French Research and Higher Education Ministry) as a collection of samples that may be used for research activities including viral quasispecies genetic characterization (Number DC-2010-1197, Collection Number 4). Access to personal data was limited to the patient age.

### Viral Genome Amplification

Viral RNA was extracted from 140 μL of respiratory specimens with Qiagen QIAamp Viral RNA kit (Cat# 52904). Ten microliters of purified RNA were then reverse transcribed with Agilent AccuScript High Fidelity 1^st^ strand cDNA Synthesis kit (Cat# 200820) using Uni1 (5′-AGCRAAAGCAGG-3′) primer. The viral genome was then amplified by PCR using Thermo Scientific Phusion High-Fidelity DNA Polymerase (Cat# F-530). All reactions were performed according to the manufacturers’ instructions. Twelve PCRs were designed to cover as much as possible of the coding regions of the eight genomic segments (primer sequences and specific annealing temperature and elongation time conditions are available in Supplementary Table 6).

### Deep Sequencing

After purification with Macherey-Nagel Nucleospin Gel and PCR Clean-up kit (Cat# 740609), the PCR products were quantified with Invitrogen Quant-iT Picogreen dsDNA Assay Kit (Cat# P7589). For each sample, two pools were prepared with the 12 PCRs covering the genome, fragmented (New England BioLabs NEBNext dsDNA Fragmentase, Cat# M0348), multiplexed, sequenced with Illumina cBot and GAIIX technology (Illumina TruSeq SR Cluster Kit v2-cBot-GA, Cat# GD-300-2001; Illumina TruSeq SBS Kit v5-GA, Cat# FC-105-5001), and analyzed with established deep sequencing data analysis tools and in-house scripts. Briefly, clipping was performed using the Galaxy tools (Mareuil et al., 2017), removing common adapter and other contaminants and trimming low quality bases (Phred < 30). Clipped reads were aligned to sequences of sH1N1 A/New Caledonia/20/1999 (GISAID Accession Nos. EPI_ISL_649) or sH3N2 A/Brisbane/10/2007 (GISAID Accession Nos. EPI_ISL_25019) or pH1N1 A/California/7/2009 (GISAID Accession Nos. EPI_ISL_159427) as reference, to extract the consensus viral sequence for each sample. For the ViVAN pipeline (Isakov et al., 2015), clipped reads of a given sample were aligned to the consensus sequence of that sample using Burrows Wheeler Aligner BWA v0.5.9 (Li and Durbin, 2009), then alignments were processed using SAMTools (Li et al., 2009) to obtain a pileup of the called bases at each position and statistically significant variants were identified above the background noise due to sequencing error, in every sufficiently covered site (>100×). In parallel, the clipped reads were aligned to the consensus sequence using Bowtie Aligner (Langmead et al., 2009) and significant variants were identified with LoFreq (Wilm et al., 2012).

### Statistical Analysis

Correlations were evaluated using Spearman non-parametric tests (Kornbrot, 2014). Differences in global mutation frequency distributions between two groups (pairwise comparisons) were compared by the two-tailed Mann–Whitney–Wilcoxon test, a non-parametric test based on rank-sums (Moses, 2014).

## Data Availability

The cleaned reads (as FASTQ files) were deposited in the European Nucleotide Archive database for NGS sequences https://www.ebi.ac.uk/ena; accession number ERP012790). In addition, the original reads (prior to cleaning) will be made available by the authors, without undue reservation, to any qualified researcher.

## Author Contributions

CB, SW, and MV conceptualized the study. CB and HB performed the template preparations and NGS sequencing. LJ, OI, GC, and NS designed the softwares and performed the bio-informatics analyses with the Variant Callers. VE gathered the resources. CB contributed to formal analysis and visualization of the data, and wrote the original draft of the manuscript. CB, SW, and MV wrote, reviewed, and edited the manuscript. SW, MV, and NS acquired the funding.

## Conflict of Interest Statement

The authors declare that the research was conducted in the absence of any commercial or financial relationships that could be construed as a potential conflict of interest.

## Footnotes

1 https://www.ebi.ac.uk/ena

## References

[B1] Acevedo A. Andino R. (2014). Library preparation for highly accurate population sequencing of RNA viruses. *Nat. Protoc.* 9 1760–1769. 10.1038/nprot.2014.118 24967624 PMC4418788

[B2] Barr I. G. Russell C. Besselaar T. G. Cox N. J. Daniels R. S. Donis R. (2014). WHO recommendations for the viruses used in the 2013-2014 Northern Hemisphere influenza vaccine: epidemiology, antigenic and genetic characteristics of influenza A(H1N1)pdm09, A(H3N2) and B influenza viruses collected from October 2012 to January 2013. *Vaccine* 32 4713–4725. 10.1016/j.vaccine.2014.02.014 24582632

[B3] Beerenwinkel N. Gunthard H. F. Roth V. Metzner K. J. (2012). Challenges and opportunities in estimating viral genetic diversity from next-generation sequencing data. *Front. Microbiol.* 3:329. 10.3389/fmicb.2012.00329 22973268 PMC3438994

[B4] Briat A. Dulioust E. Galimand J. Fontaine H. Chaix M. L. Letur-Konirsch H. (2005). Hepatitis C virus in the semen of men coinfected with HIV-1: prevalence and origin. *AIDS* 19 1827–1835. 10.1097/01.aids.0000189847.98569.2d 16227790

[B5] Chen H. Wen X. To K. K. Wang P. Tse H. Chan J. F. (2010). Quasispecies of the D225G substitution in the hemagglutinin of pandemic influenza A(H1N1) 2009 virus from patients with severe disease in Hong Kong, China. *J. Infect. Dis.* 201 1517–1521. 10.1086/652661 20367331

[B6] Debbink K. McCrone J. T. Petrie J. G. Truscon R. Johnson E. Mantlo E. K. (2017). Vaccination has minimal impact on the intrahost diversity of H3N2 influenza viruses. *PLoS Pathog.* 13:e1006194. 10.1371/journal.ppat.1006194 28141862 PMC5302840

[B7] Dinis J. M. Florek N. W. Fatola O. O. Moncla L. H. Mutschler J. P. Charlier O. K. (2016). Deep sequencing reveals potential antigenic variants at low frequencies in influenza A virus-infected humans. *J. Virol.* 90 3355–3365. 10.1128/JVI.03248-15 26739054 PMC4794676

[B8] Domingo E. (2006). Quasispecies: concept and implication for virology. *Curr. Top. Microbiol. Immunol.* 299 10.1007/b137531 PMC7120838 16568896

[B9] Drake J. W. Holland J. J. (1999). Mutation rates among RNA viruses. *Proc. Natl. Acad. Sci. U.S.A.* 96 13910–13913. 10.1073/Pnas.96.24.13910 10570172 PMC24164

[B10] Fisher R. van Zyl G. U. Travers S. A. Kosakovsky Pond S. L. Engelbrech S. Murrell B. (2012). Deep sequencing reveals minor protease resistance mutations in patients failing a protease inhibitor regimen. *J. Virol.* 86 6231–6237. 10.1128/jvi.06541-11 22457522 PMC3372173

[B11] Fordyce S. L. Bragstad K. Pedersen S. S. Jensen T. G. Gahrn-Hansen B. Daniels R. (2013). Genetic diversity among pandemic 2009 influenza viruses isolated from a transmission chain. *Virol. J.* 10:116. 10.1186/1743-422x-10-116 23587185 PMC3639878

[B12] Ghedin E. Holmes E. C. DePasse J. V. Pinilla L. T. Fitch A. Hamelin M.-E. (2012). Presence of oseltamivir-resistant pandemic A/H1N1 minor variants before drug therapy with subsequent selection and transmission. *J. Infect. Dis.* 206 1504–1511. 10.1093/infdis/jis571 22966122 PMC3475640

[B13] Ghedin E. Laplante J. DePasse J. Wentworth D. E. Santos R. P. Lepow M. L. (2011). Deep sequencing reveals mixed infection with 2009 pandemic Influenza A (H1N1) virus strains and the emergence of oseltamivir resistance. *J. Infect. Dis.* 203 168–174. 10.1093/infdis/jiq040 21288815 PMC3071067

[B14] Hoper D. Hoffmann B. Beer M. (2011). A comprehensive deep sequencing strategy for full-length genomes of influenza A. *PLoS One* 6:e19075. 10.1371/journal.pone.0019075 21559493 PMC3084732

[B15] Isakov O. Borderia A. V. Golan D. Hamenahem A. Celniker G. Yoffe L. (2015). Deep sequencing analysis of viral infection and evolution allows rapid and detailed characterization of viral mutant spectrum. *Bioinformatics* 31 2141–2150. 10.1093/bioinformatics/btv101 25701575 PMC4481840

[B16] Jagger B. W. Wise H. M. Kash J. C. Walters K. A. Wills N. M. Xiao Y. L. (2012). An overlapping protein-coding region in influenza A virus segment 3 modulates the host response. *Science (80-)* 337 199–204. 10.1126/science.1222213 22745253 PMC3552242

[B17] Kampmann M. L. Fordyce S. L. Avila-Arcos M. C. Rasmussen M. Willerslev E. Nielsen L. P. (2011). A simple method for the parallel deep sequencing of full influenza A genomes. *J. Virol. Methods* 178 243–248. 10.1016/j.jviromet.2011.09.001 21946287

[B18] Klein E. Y. Serohijos A. W. Choi J. M. Shakhnovich E. I. Pekosz A. (2014). Influenza A H1N1 pandemic strain evolution–divergence and the potential for antigenic drift variants. *PLoS One* 9:e93632. 10.1371/journal.pone.0093632 24699432 PMC3974778

[B19] Kornbrot D. (2014). *Spearman’s Rho*: Wiley StatsRef Stat. Ref. Online. Hoboken, NJ: John Wiley & Sons.

[B20] Kuroda M. Katano H. Nakajima N. Tobiume M. Ainai A. Sekizuka T. (2010). Characterization of quasispecies of pandemic 2009 influenza A virus (A/H1N1/2009) by de novo sequencing using a next-generation DNA sequencer. *PLoS One* 5:e10256. 10.1371/journal.pone.0010256 20428231 PMC2859049

[B21] Langmead B. Trapnell C. Pop M. Salzberg S. L. (2009). Ultrafast and memory-efficient alignment of short DNA sequences to the human genome. *Genome Biol.* 10:R25. 10.1186/gb-2009-10-3-r25 19261174 PMC2690996

[B22] Lauring A. S. Andino R. (2010). Quasispecies theory and the behavior of RNA viruses. *PLoS Pathog* 6:e1001005. 10.1371/journal.ppat.1001005 20661479 PMC2908548

[B23] Lee H. K. Lee C. K. Tang J. W.-T. Loh T. P. Koay E. S.-C. (2016). Contamination-controlled high-throughput whole genome sequencing for influenza A viruses using the MiSeq sequencer. *Sci. Rep.* 6:33318. 10.1038/srep33318 27624998 PMC5022032

[B24] Li H. Durbin R. (2009). Fast and accurate short read alignment with Burrows-Wheeler transform. *Bioinformatics* 25 1754–1760. 10.1093/bioinformatics/btp324 19451168 PMC2705234

[B25] Li H. Handsaker B. Wysoker A. Fennell T. Ruan J. Homer N. (2009). The sequence alignment/map format and SAMtools. *Bioinformatics* 25 2078–2079. 10.1093/bioinformatics/btp352 19505943 PMC2723002

[B26] Li J. Z. Paredes R. Ribaudo H. J. Kozal M. J. Svarovskaia E. S. Johnson J. A. (2013). Impact of minority nonnucleoside reverse transcriptase inhibitor resistance mutations on resistance genotype after virologic failure. *J. Infect. Dis.* 207 893–897. 10.1093/infdis/jis925 23264671 PMC3571444

[B27] Lin Z. Farooqui A. Li G. Wong G. K. Mason A. L. Banner D. (2014). Next-generation sequencing and bioinformatic approaches to detect and analyze influenza virus in ferrets. *J. Infect Dev. Ctries* 8 498–509. 10.3855/jidc.3749 24727517

[B28] Mareuil F. Doppelt-Azeroual O. Ménager H. (2017). “A public Galaxy platform at Pasteur used as an execution engine for web services” in *Poster at the F1000Research* 6:1030 10.7490/F1000RESEARCH.1114334.1

[B29] McCrone J. T. Woods R. J. Martin E. T. Malosh R. E. Monto A. S. Lauring A. S. (2017). Stochastic processes dominate the within and between host evolution of influenza virus. *Elite* 7:e35962. 10.1101/176362 29683424 PMC5933925

[B30] Meijer A. Lackenby A. Hungnes O. Lina B. van der Werf S. Schweiger B. (2009). Oseltamivir-resistant influenza Virus A (H1N1), Europe, 2007–08 Season. *Emerg. Infect. Dis.* 15 552–560. 10.3201/eid1504.081280 19331731 PMC2671453

[B31] Meinel D. M. Heinzinger S. Eberle U. Ackermann N. Schönberger K. Sing A. (2018). Whole genome sequencing identifies influenza A H3N2 transmission and offers superior resolution to classical typing methods. *Infection* 46 69–76. 10.1007/s15010-017-1091-3 29086356

[B32] Molinari N. A. Ortega-Sanchez I. R. Messonnier M. L. Thompson W. W. Wortley P. M. Weintraub E. (2007). The annual impact of seasonal influenza in the US: measuring disease burden and costs. *Vaccine* 25 5086–5096. 10.1016/j.vaccine.2007.03.046 17544181

[B33] Moncla L. H. Ross T. M. Dinis J. M. Weinfurter J. T. Mortimer T. D. Schultz-Darken N. (2013). A novel nonhuman primate model for influenza transmission. *PLoS One* 8:e78750. 10.1371/journal.pone.0078750 24244352 PMC3828296

[B34] Monne I. Fusaro A. Nelson M. I. Bonfanti L. Mulatti P. Hughes J. (2014). Emergence of a highly pathogenic avian influenza virus from a low-pathogenic progenitor. *J. Virol.* 88 4375–4388. 10.1128/JVI.03181-13 24501401 PMC3993777

[B35] Moses L. E. (2014). *Wilcoxon-Mann-Whitney Test: Definition and Example: Wiley StatsRef Stat. Ref. Online.* Hoboken, NJ: John Wiley & Sons 10.1002/9781118445112.stat06898

[B36] Munster V. J. Baas C. Lexmond P. Waldenstrom J. Wallensten A. Fransson T. (2007). Spatial, temporal, and species variation in prevalence of influenza A viruses in wild migratory birds. *PLoS Pathog.* 3:e61. 10.1371/journal.ppat.0030061 17500589 PMC1876497

[B37] Muramoto Y. Noda T. Kawakami E. Akkina R. Kawaoka Y. (2013). Identification of novel influenza A virus proteins translated from PA mRNA. *J. Virol.* 87 2455–2462. 10.1128/JVI.02656-12 23236060 PMC3571384

[B38] Nakamura K. Oshima T. Morimoto T. Ikeda S. Yoshikawa H. Shiwa Y. (2011). Sequence-specific error profile of Illumina sequencers. *Nucleic Acids Res.* 39:e90. 10.1093/nar/gkr344 21576222 PMC3141275

[B39] Nasu A. Marusawa H. Ueda Y. Nishijima N. Takahashi K. Osaki Y. (2011). Genetic heterogeneity of hepatitis C virus in association with antiviral therapy determined by ultra-deep sequencing. *PLoS One* 6:e24907. 10.1371/journal.pone.0024907 21966381 PMC3178558

[B40] Neumann G. Noda T. Kawaoka Y. (2009). Emergence and pandemic potential of swine-origin H1N1 influenza virus. *Nature* 459 931–939. 10.1038/nature08157 19525932 PMC2873852

[B41] Nishijima N. Marusawa H. Ueda Y. Takahashi K. Nasu A. Osaki Y. (2012). Dynamics of hepatitis B virus quasispecies in association with nucleos(t)ide analogue treatment determined by ultra-deep sequencing. *PLoS One* 7:e35052. 10.1371/journal.pone.0035052 22523569 PMC3327662

[B42] Ortega-Sanchez I. R. Molinari N. A. Fairbrother G. Szilagyi P. G. Edwards K. M. Griffin M. R. (2012). Indirect, out-of-pocket and medical costs from influenza-related illness in young children. *Vaccine* 30 4175–4181. 10.1016/j.vaccine.2012.04.057 22546332

[B43] Paranjpe S. Craigo J. Patterson B. Ding M. Barroso P. Harrison L. (2002). Subcompartmentalization of HIV-1 quasispecies between seminal cells and seminal plasma indicates their origin in distinct genital tissues. *AIDS Res. Hum. Retrovir.* 18 1271–1280. 10.1089/088922202320886316 12487815

[B44] Parvin J. D. Moscona A. Pan W. T. Leider J. M. Palese P. (1986). Measurement of the mutation rate of animal viruses: influenza A virus and poliovirus type 1. *J. Virol.* 59 377–383. 3016304 10.1128/jvi.59.2.377-383.1986 PMC253087

[B45] Pichon M. Gaymard A. Josset L. Valette M. Millat G. Lina B. (2017). Characterization of oseltamivir-resistant influenza virus populations in immunosuppressed patients using digital-droplet PCR: comparison with qPCR and next generation sequencing analysis. *Antiviral Res.* 145 160–167. 10.1016/J.ANTIVIRAL.2017.07.021 28780426

[B46] Pizzorno A. Abed Y. Plante P. L. Carbonneau J. Baz M. Hamelin M. E. (2014). Evolution of oseltamivir resistance mutations in Influenza A(H1N1) and A(H3N2) viruses during selection in experimentally infected mice. *Antimicrob. Agents Chemother.* 58 6398–6405. 10.1128/aac.02956-14 25114143 PMC4249448

[B47] Poon L. L. M. Song T. Rosenfeld R. Lin X. Rogers M. B. Zhou B. (2016). Quantifying influenza virus diversity and transmission in humans. *Nat. Genet.* 48 195–200. 10.1038/ng.3479 26727660 PMC4731279

[B48] Quinones-Mateu M. E. Avila S. Reyes-Teran G. Martinez M. A. (2014). Deep sequencing: becoming a critical tool in clinical virology. *J. Clin. Virol.* 61 9–19. 10.1016/j.jcv.2014.06.013 24998424 PMC4119849

[B49] Ren X. Yang F. Hu Y. Zhang T. Liu L. Dong J. (2013). Full genome of influenza A (H7N9) virus derived by direct sequencing without culture. *Emerg. Infect. Dis.* 19 1881–1884. 10.3201/eid1911.130664 24206919 PMC3837655

[B50] Rodriguez C. Chevaliez S. Bensadoun P. Pawlotsky J. M. (2013). Characterization of the dynamics of hepatitis B virus resistance to adefovir by ultra-deep pyrosequencing. *Hepatology* 58 890–901. 10.1002/hep.26383 23505208

[B51] Rogers M. B. Song T. Sebra R. Greenbaum B. D. Hamelin M. E. Fitch A. (2015). Intrahost dynamics of antiviral resistance in influenza a virus reflect complex patterns of segment linkage, reassortment, and natural selection. *MBio* 6:e02464-14. 10.1128/mBio.02464-14 25852163 PMC4453542

[B52] Rutvisuttinunt W. Chinnawirotpisan P. Simasathien S. Shrestha S. K. Yoon I.-K. Klungthong C. (2013). Simultaneous and complete genome sequencing of influenza A and B with high coverage by Illumina MiSeq platform. *J. Virol. Methods* 193 394–404. 10.1016/J.JVIROMET.2013.07.001 23856301

[B53] Sanjuan R. Nebot M. R. Chirico N. Mansky L. M. Belshaw R. (2010). Viral mutation rates. *J. Virol.* 84 9733–9748. 10.1128/JVI.00694-10 20660197 PMC2937809

[B54] Shaw M. L. Palese P. (2013). “Orthomyxoviridae,” in *Fields Virology* 6th Edn eds Knipe D. M. Howley P. M. (Philadelphia: Lippincott Williams & Wilkins) 1151–1185.

[B55] Simen B. B. Simons J. F. Hullsiek K. H. Novak R. M. Macarthur R. D. Baxter J. D. (2009). Low-abundance drug-resistant viral variants in chronically HIV-infected, antiretroviral treatment-naive patients significantly impact treatment outcomes. *J. Infect. Dis.* 199 693–701. 10.1086/596736 19210162

[B56] Stapleford K. A. Coffey L. L. Lay S. Borderia A. V Duong V. Isakov O. (2014). Emergence and transmission of arbovirus evolutionary intermediates with epidemic potential. *Cell Host Microbe* 15 706–716. 10.1016/j.chom.2014.05.008 24922573

[B57] Steinhauer D. A. de la Torre J. C. Holland J. J. (1989a). High nucleotide substitution error frequencies in clonal pools of vesicular stomatitis virus. *J. Virol.* 63 2063–2071. 2539502 10.1128/jvi.63.5.2063-2071.1989 PMC250622

[B58] Steinhauer D. A. de la Torre J. C. Meier E. Holland J. J. (1989b). Extreme heterogeneity in populations of vesicular stomatitis virus. *J. Virol.* 63 2072–2080. 2539503 10.1128/jvi.63.5.2072-2080.1989 PMC250623

[B59] Sutton T. C. Finch C. Shao H. Angel M. Chen H. Capua I. (2014). Airborne transmission of highly pathogenic H7N1 influenza virus in ferrets. *J. Virol.* 88 6623–6635. 10.1128/JVI.02765-13 24696487 PMC4054360

[B60] Svarovskaia E. S. Martin R. McHutchison J. G. Miller M. D. Mo H. (2012). Abundant drug-resistant NS3 mutants detected by deep sequencing in hepatitis C virus-infected patients undergoing NS3 protease inhibitor monotherapy. *J. Clin. Microbiol.* 50 3267–3274. 10.1128/jcm.00838-12 22837328 PMC3457457

[B61] Téllez-Sosa J. Rodríguez M. H. Gómez-Barreto R. E. Valdovinos-Torres H. Hidalgo A. C. Cruz-Hervert P. (2013). Using high-throughput sequencing to leverage surveillance of genetic diversity and oseltamivir resistance: a pilot study during the 2009 Influenza A(H1N1) pandemic. *PLoS One* 8:e67010. 10.1371/journal.pone.0067010 23843978 PMC3699567

[B62] Thyagarajan B. Bloom J. D. (2014). The inherent mutational tolerance and antigenic evolvability of influenza hemagglutinin. *Elife* 3:e03300. 10.7554/eLife.03300 25006036 PMC4109307

[B63] Tong S. Zhu X. Li Y. Shi M. Zhang J. Bourgeois M. (2013). New world bats harbor diverse influenza A viruses. *PLoS Pathog.* 9:e1003657. 10.1371/journal.ppat.1003657 24130481 PMC3794996

[B64] Tscherne D. M. Garcia-Sastre A. (2011). Virulence determinants of pandemic influenza viruses. *J. Clin. Invest.* 121 6–13. 10.1172/jci44947 21206092 PMC3007163

[B65] Watson S. J. Welkers M. R. Depledge D. P. Coulter E. Breuer J. M. de Jong M. D. (2013). Viral population analysis and minority-variant detection using short read next-generation sequencing. *Philos. Trans. R Soc. L B Biol. Sci.* 368:20120205. 10.1098/rstb.2012.0205 23382427 PMC3678329

[B66] Welkers M. R. A. Jonges M. Jeeninga R. E. Koopmans M. P. G. de Jong M. D. (2015). Improved detection of artifactual viral minority variants in high-throughput sequencing data. *Front. Microbiol.* 5:804. 10.3389/fmicb.2014.00804 25657642 PMC4302989

[B67] Willerth S. M. Pedro H. A. Pachter L. Humeau L. M. Arkin A. P. Schaffer D. V. (2010). Development of a low bias method for characterizing viral populations using next generation sequencing technology. *PLoS One* 5:e13564. 10.1371/journal.pone.0013564 21042592 PMC2962647

[B68] Wilm A. Aw P. P. K. Bertrand D. Yeo G. H. T. Ong S. H. Wong C. H. (2012). LoFreq: a sequence-quality aware, ultra-sensitive variant caller for uncovering cell-population heterogeneity from high-throughput sequencing datasets. *Nucleic Acids Res.* 40 11189–11201. 10.1093/nar/gks918 23066108 PMC3526318

[B69] Wise H. M. Barbezange C. Jagger B. W. Dalton R. M. Gog J. R. Curran M. D. (2011). Overlapping signals for translational regulation and packaging of influenza A virus segment 2. *Nucleic Acids Res.* 39 7775–7790. 10.1093/nar/gkr487 21693560 PMC3177217

[B70] Xue K. S. Moncla L. H. Bedford T. Bloom J. D. (2018). Within-host evolution of human influenza virus. *Trends Microbiol.* 26 781–793. 10.1016/J.TIM.2018.02.007 29534854 PMC6097882

[B71] Yamayoshi S. Watanabe M. Goto H. Kawaoka Y. (2015). Identification of A novel viral protein expressed from the PB2 segment of influenza A virus. *J. Virol.* 90 444–456. 10.1128/jvi.02175-15 26491155 PMC4702538

[B72] Yang J. R. Lin Y. C. Huang Y. P. Su C. H. Lo J. Ho Y. L. (2011). Reassortment and mutations associated with emergence and spread of oseltamivir-resistant seasonal influenza A/H1N1 viruses in 2005-2009. *PLoS One* 6:e18177. 10.1371/journal.pone.0018177 21483816 PMC3069057

[B73] Zhao J. Liu J. Vemula S. V. Lin C. Tan J. Ragupathy V. (2016). Sensitive detection and simultaneous discrimination of influenza A and B viruses in nasopharyngeal swabs in a single assay using next-generation sequencing-based diagnostics. *PLoS One* 11:e0163175. 10.1371/journal.pone.0163175 27658193 PMC5033603

