# Supplementary figures and images for: Seasonal Genetic Drift of Human Influenza A Virus Quasispecies Revealed by Deep Sequencing
Source: Front Microbiol. 2018 Oct 31;9:2596. doi: 10.3389/fmicb.2018.02596 (PMC6220372; doi:10.3389/fmicb.2018.02596)

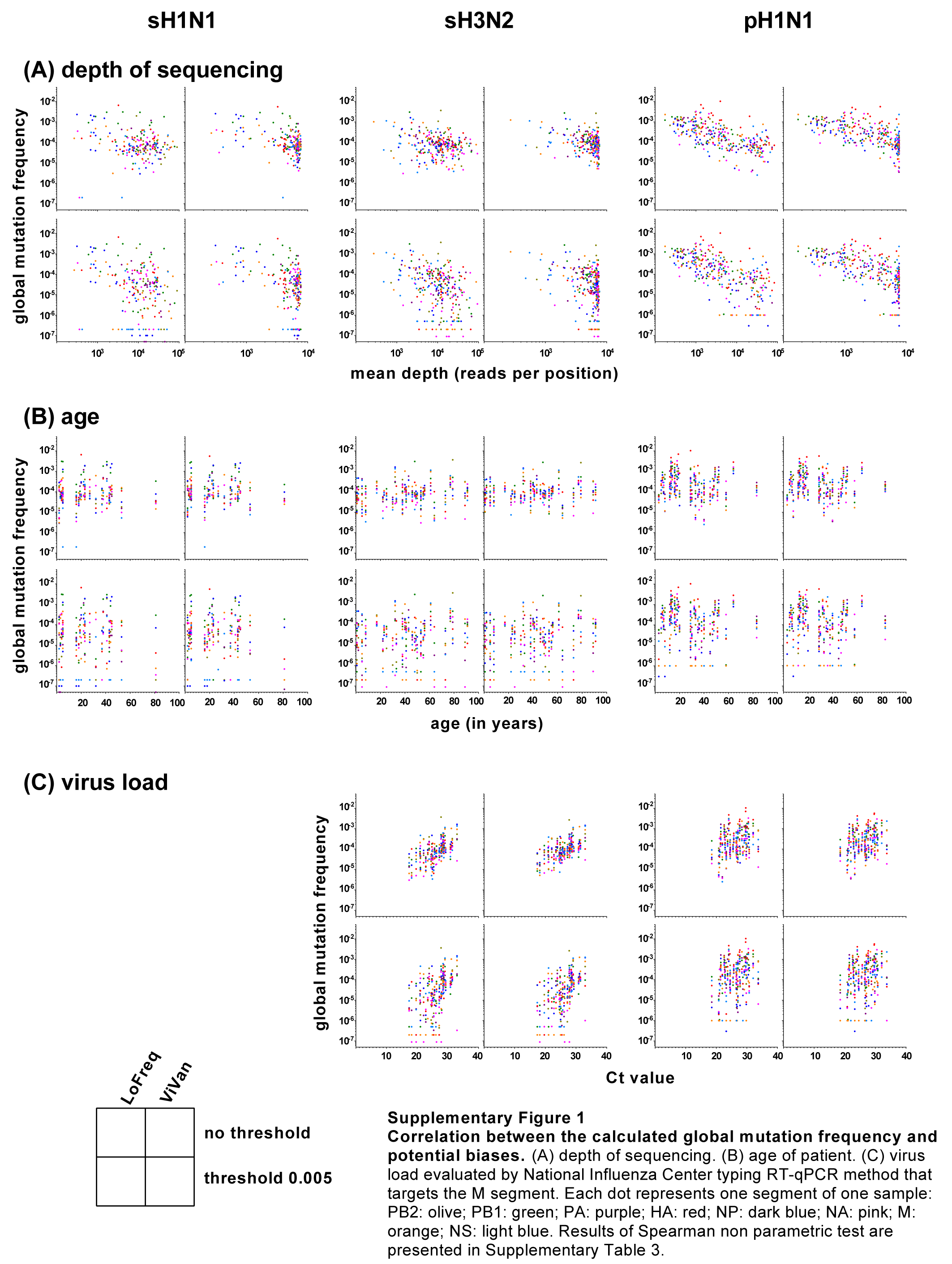

Supplement: Supplementary file 7 [file Image_1.TIF]
